# Competition among Flavescence Dorée Phytoplasma Strains in the Experimental Insect Vector *Euscelidius variegatus*

**DOI:** 10.3390/insects14070575

**Published:** 2023-06-23

**Authors:** Marika Rossi, Luciana Galetto, Nicola Bodino, Jessica Beltramo, Silvia Gamalero, Mattia Pegoraro, Domenico Bosco, Cristina Marzachì

**Affiliations:** 1 Istituto per la Protezione Sostenibile delle Piante, Consiglio Nazionale delle Ricerche, IPSP-CNR, Strada delle Cacce 73, 10135 Torino, Italy; marika.rossi@ipsp.cnr.it (M.R.); nicola.bodino@ipsp.cnr.it (N.B.); jessicabeltramo7@gmail.com (J.B.); silvia.gamalero@outlook.it (S.G.); m.pegoraro@inrim.it (M.P.); domenico.bosco@unito.it (D.B.); cristina.marzachi@ipsp.cnr.it (C.M.); 2 Dipartimento di Scienze Agrarie, Forestali ed Alimentari DISAFA, Università degli Studi di Torino, Largo Paolo Braccini 2, 10095 Grugliasco, Italy; 3 Dipartimento di Scienze dell’Ambiente e della Vita, Università del Piemonte Orientale “Amedeo Avogadro”, Viale Teresa Michel 11, 15121 Alessandria, Italy; 4 Metrologia dei Materiali Innovativi e Scienze della Vita, Istituto Nazionale di Ricerca Metrologica, INRiM, Strada delle Cacce 91, 10135 Torino, Italy

**Keywords:** grapevine Flavescence dorée, leafhopper, Hemiptera, Cicadellidae, 16SrV-C and -D ribosomal groups

## Abstract

**Simple Summary:**

Flavescence dorée (FD) is a serious disease of grapevine, spread in Europe and caused by phytoplasmas. They are uncultivable bacteria, transmitted from plant to plant by hemipteran insects (mainly leafhoppers) and classified according to their genetic traits. Two different phytoplasma strains are associated with the disease, namely FD-C and FD-D. The former outcompetes the latter during the infection of an experimental plant host (periwinkle), although the latter is more abundant in vineyards. Mixed infections are rare in the field. Here, competition between FD-C and FD-D pathogen strains was investigated during the infection of the laboratory insect vector *Euscelidius variegatus* (Hemiptera: Cicadellidae). Although insects were forced to acquire both strains, single infection, irrespective of the strain type, was more frequent than expected, probably due to competition among strains. Management of the disease mainly relies on the use (compulsory in some European areas) of insecticides, with evident undesirable effects on the environment and public health. Deciphering mechanisms regulating the epidemiology of FDp strains may pave the way towards the integrated management of the disease, such as by fine-tuning the treatments and identifying mild suppressor strains to outcompete the severe ones.

**Abstract:**

Phytoplasmas are plant pathogenic wall-less bacteria transmitted in a persistent propagative manner by hemipteran insects, mainly belonging to the suborder Auchenorrhyncha (Fulgoromorpha and Cicadomorpha). Flavescence dorée (FD) is a quarantine disease of grapevine, causing great damage to European viticulture and associated with phytoplasmas belonging to 16SrV-C (FD-C) and -D (FD-D) subgroups. FD-C and FD-D strains share similar pathogenicity, but mixed infections are rare in nature. To investigate the competition among FDp strains, specimens of the laboratory vector *Euscelidius variegatus* (Hemiptera: Cicadellidae) were forced to acquire both phytoplasma haplotypes upon feeding on FD-C- and FD-D-infected plants or after the injection of both strains. The pathogen colonization of insect bodies and heads was monitored with multiplex qPCR, and the efficiencies of phytoplasma transmission were estimated. Single infection, irrespective of strain type, was more frequent than expected, indicating that competition among FD strains occurs. Hypotheses of competition for resources and/or host active sites or the direct antibiosis of one strain against the other are discussed, based on the genetic complexity of FDp populations and on the high genome variability of the FD-D strain. As FD management still mainly relies on insecticides against vectors, the characterization of FDp haplotypes and the description of their epidemiology also have practical implications.

## 1. Introduction

Phytoplasmas are plant pathogenic bacteria that invade the phloem elements of the host plants and colonize the bodies of insect vectors. They are associated with hundreds of plant diseases worldwide and are responsible for severe economic losses to important crops [1,2]. Although phytoplasmas represent a well-defined monophyletic clade in the family Acholeplasmataceae, they are still classified as indefinite taxa due to severe difficulties impairing in vitro cultivation [3]. Their classification is based on 16S rRNA gene and the “*Candidatus* species” concept is applied for well-characterized phytoplasmas [4]. To our knowledge, there are 49 officially described “*Candidatus* Phytoplasma species” nowadays [5].

The 16SrV phytoplasma phylogenetic group comprises six phytoplasma subgroups, four of which are described as “*Candidatus* species”, namely “*Ca.* P. ulmi” (16SrV-A) [6], “*Ca*. P. ziziphin” (16SrV-B) [7], “*Ca.* P. rubi” (16SrV-E) [8], and “*Ca.* P. balanitae” (16SrV-F) [9]. The remaining two subgroups, 16SrV-C and -D, have been proposed for the not formally described “*Ca.* P. vitis” [4,10]. They include the phytoplasma associated with the grapevine Flavescence dorée (FDp), which is a quarantine pest and a major threat to European viticulture [11,12,13,14]. Analyses of polymorphisms in ribosomal and nonribosomal elements highlighted the genetic differences between the closely related genotypes clustering in 16SrV-C and -D subgroups [15,16,17,18,19]. Both strains (hereafter FD-C and FD-D) share similar pathogenicity and symptomatology in infected grapevines. However, the FD-D subgroup is now present in more than 70% of the field disease cases in Piedmont (Italy) where it has replaced FD-C, the prevalent causal agent of the epidemics in 1998 [20]. On the other hand, in the same area, the prevalence of the FD-C strain is higher in plants than in insects [18,21]. The subgroup 16SrV-D is the most prevalent strain also in other European wine areas, such as France, Spain, as well as Lombardy and Veneto (Italy) [15,17,22,23].

Phytoplasma vectors are hemipteran insects belonging to the suborder Auchenorrhyncha (Fulgoromorpha and Cicadomorpha) and to the family Psyllidae (suborder Sternorrhyncha) [24,25]. These wall-less bacteria are transmitted in a persistent propagative manner [26] and several studies have suggested the involvement of specific molecular interactions in transmission specificity with insect hosts [27,28,29,30,31,32,33]. The leafhopper *Scaphoideus titanus* Ball (Cicadellidae: Deltocephalinae) has major epidemiological significance for FD disease in European vineyards [13,34]. Other insect species are competent vectors, but they play minor roles in spreading FD, being associated with different epidemiological routes [19]. The polyvoltine leafhopper *Euscelidius variegatus* Kirschbaum (Cicadellidae: Deltocephalinae) is closely related with *S. titanus* and is commonly used as a laboratory FDp vector, being able to efficiently transmit the phytoplasma to broad beans [35,36,37].

It is noteworthy that (i) both FD-C and FD-D strains are transmitted by *S. titanus* and *E. variegatus* [38], (ii) mixed FD-C + FD-D infections are very rare under field conditions both in plants and in insects [18,21], and (iii) FD-C outcompetes FD-D during the infection of the experimental plant host *Catharanthus roseus* (periwinkle) [39]. In order to decipher strain competition in insects, the condition of mixed FD-C and FD-D was investigated in the vector species *E. variegatus* by forcing insects to naturally acquire both phytoplasmas after feeding on FD-C and FD-D infected plants and after the injection of both strains mixed together. The pathogen colonization of insect bodies (thorax + abdomen) and heads (including salivary glands) was separately monitored with multiplex qPCR analysis, and the efficiencies of phytoplasma transmission to the test plants and to artificial feeding media were estimated. The occurrence of competition between FDp strains in insects would help in understanding disease epidemiology and possibly improving control strategies.

## 2. Materials and Methods

### 2.1. Plants, Insects, and Phytoplasma Strains

Plants of oat (*Avena sativa*) and broad bean (*Vicia faba* “Agua-dulce Supersimonia”) were grown from seed in pots in greenhouse, at 24 ± 2 °C, and used 2 weeks after sowing to rear healthy colonies of the leafhopper *E. variegatus* (*A. sativa*) or as host plants to maintain the FDp isolates (*V. faba*).

*Euscelidius variegatus* was originally collected in Piedmont and continuously reared on oats inside plastic and nylon cages in growth chambers at 20–25 °C with a L16:D8 photoperiod.

Flavescence dorée phytoplasma strains “FD-C Piedmont” [40] and “FD-D CRA AT” [41] were originally isolated in Piedmont and then routinely maintained on *V. faba* plants with sequential transmission by *E. variegatus* [37].

### 2.2. Mixed Acquisition by Feeding on FD-C- and FD-D-Infected Plants

To determine insects’ ability to contemporarily acquire both phytoplasma strains, nearly 150 healthy 4th- and 5th-instar *E. variegatus* nymphs were caged together and allowed to feed for two weeks on four FDp-infected broad bean source plants, two infected with the FD-C strain and the other two with the FD-D one. To guarantee an equivalent source of inoculum for each strain, phytoplasma loads were measured in each source plant using qPCR [39] just before the acquisition phase, and only plants displaying similar pathogen loads were selected and used for mixed acquisition experiments. To increase the probability of random feeding on plants infected with different phytoplasma strains, during the acquisition access period (AAP), the insects were disturbed daily by gently shaking the source plants. At the end of AAP, infected broad beans were removed and insects were fed on fresh oats (immune to phytoplasmas) for an additional two weeks to complete latency period (LP). This experimental set up was named “Mixed acquisition by feeding”, and it was repeated twice.

### 2.3. Mixed Acquisition via Abdominal Microinjection of FD-C and FD-D Suspension

To determine insects’ ability to sustain a mixed infection, newly emerged *E. variegatus* adults were injected with phytoplasma suspensions containing an equivalent amount (measured with qPCR) of both pathogen strains mixed together. To obtain coeval newly emerged *E. variegatus* adults, about two weeks before each experiment, the required numbers of 4th- and 5th-instar nymphs were caged together on oats, separately from the main rearing, and then used for abdominal microinjection once they emerged as adults. To obtain phytoplasma suspension, groups of 30 infected adults (FD-C and FD-D) were separately ground in a tissue grinder with 900 μL of extraction buffer (300 mM glycine, 30 mM MgCl_2_, pH 8.0) [42]. The homogenates were clarified using centrifugation for 10 min at 800× *g* and the supernatants were filtered through 0.45 μm sterile filters. The above steps were performed at 4 °C. The phytoplasma suspensions (crude phytoplasma extracts) were maintained on ice and used within the day of preparation. Phytoplasma load was measured with qPCR on crude extracts as detailed below and then diluted to obtain the same amount of each phytoplasma strain. The mixed FD-C/FD-D suspension was injected into hoppers with glass microinjection needles made with a needle puller device (about 50 insects/treatment). Newly emerged adults of healthy *E. variegatus* were anaesthetized with CO_2_ and injected between two abdominal segments. Injected leafhoppers were caged on oat for three weeks of LP. This experimental set up was named “Mixed acquisition by microinjection”, and it was repeated twice.

### 2.4. Phytoplasma Transmission following “Mixed Acquisition by Feeding”

To assess insects’ capability to transmit both FDp strains, insects fed on infected plants were singly isolated on healthy broad bean plants for a one-week inoculation access period (IAP). To exclude possible competition between pathogen strains in the plant following the IAP [39], a subset of insects were also singly fed for 48 h on artificial feeding sachets to determine phytoplasma strains secreted into feeding media. Preliminary assays (not shown) were useful to determine the best-performing conditions, which were adapted from previous protocols [43,44]. In particular, microcentrifuge tubes (2 mL) were used as insect chambers by filling the cap with 270 μL of 5% sucrose in TE (10 mM Tris (pH 8.0), 1 mM EDTA) and sealing it with Parafilm. The bottom ends of the microcentrifuge tubes were cut, an individual insect was placed in each, and the cut end was sealed with cotton wool. Each tube was kept horizontally facing a light source to attract the insects to the feeding medium.

Insects were collected at the end of IAP (on plants and on artificial media) for further analysis, plants were transferred in an insect-proof greenhouse for 40 days, and feeding media were collected for DNA extraction and PCR detection. Symptoms of phytoplasma infection on inoculated broad beans were evaluated twice a week and total DNAs were then extracted from each single inoculated plant for PCR detection at five and seven weeks after IAP. The experiment was repeated twice.

### 2.5. Insect Dissection

Insects were collected at the end of the experiments and the head and thorax + abdomen (hereafter referred to as “body”) were dissected under a stereomicroscope. The head, with salivary glands, was separated by lifting the clypeus with forceps and the two parts were separately rinsed twice in sterile phosphate-buffered saline (PBS) 1×. The heads and bodies were individually stored at –20 °C before DNA extraction. The heads and bodies from healthy insects were also dissected, stored, and analyzed as negative experimental controls.

### 2.6. DNA Extraction, Phytoplasma Diagnosis, and Quantification of Pathogen Load

Total DNA was extracted with cethyl-trimethyl-ammonium bromide (CTAB) buffer from broad bean samples (0.5–1 g of leaf tissues) as described in [45], in order to measure phytoplasma load in source-infected plants used for “Mixed acquisition by feeding” and to determine effective transmission in singly inoculated plants.

Total DNA was also extracted with CTAB buffer from the dissected heads and bodies of *E. variegatus* [46] to assess insects’ capability to acquire both FDp strains together and to sustain their infection.

Total DNA was also extracted from feeding media according to the previously optimized protocol [44]. Briefly, artificial media were collected after the feeding phase and centrifuged at 12,000× *g* for 20 min at 4 °C in order to precipitate phytoplasma cells. DNA was extracted by adding 10 μL of 0.5 M NaOH, followed by the addition of 20 μL of 1 M Tris-HCl (pH 8.0) containing 1% sodium dodecyl sulfate and 20 mM EDTA. The mixture was incubated at 65 °C for 15 min and precipitated with 2 volumes of absolute ethanol.

Plant, insect, and medium samples were resuspended in 100, 50, and 12 μL of 10 mM Tris-HCl pH 8.0, respectively. The concentration and purity of extracted total DNAs were checked with a UV–visible spectrophotometer Nanodrop 2000 (Thermofisher). The samples were diluted to 20 ng/μL and 1 μL was used as template in each replicate of qPCR reaction.

To determine pathogen loads in phytoplasma suspensions and obtain equal amounts of both strains for the injection experiments, qPCR was directly run on diluted aliquots (1:100 in sterile H_2_O) of crude phytoplasma extracts, avoiding the DNA extraction procedure. Diluted aliquots of phytoplasma suspensions were boiled for 5 min and 1 μL was used as a template in each replicate of the qPCR reaction. This procedure was performed on the same day as suspension preparation and injection.

The primer pair nrdF_F28/R121 together with the TaqMan probes nrdF-C (5′HEX-labelled) and nrdF-D (5′FAM-labelled) [39] was used to detect and quantify FDp presence via qPCR, using 1x iTaq Universal Probe Supermix (Bio-Rad) in a multiplex reaction mix of 10 μL volume. The primer pair, together with strain-specific probes targeting the FDp *nrd*F gene, are able to detect the single presence of either FD-C or FD-D strains as well as mixed infections of both isolates [39]. Final concentrations were 300 and 200 nM for primers and probes, respectively, and cycling conditions were as indicated in the original paper [39]. The samples were run in triplicate in a CFX Connect Real-Time PCR Detection System (Bio-Rad) together with healthy samples and no-template controls.

For the absolute quantification of the two FDp strains, two standard curves were obtained from recombinant pGEM plasmids harboring a fragment of the corresponding targets (pGEM-NrdF-C; pGEM-NrdF-D), serially diluted from 100 pg to 10 fg, corresponding to 10^7^ to 10 genomic units (GU) of FDp [39]. Standard curves were constructed with the CFX Manager™ Software via a linear regression analysis of the Cq value of each standard dilution replicate over the log of the number of plasmid copies present in each sample.

### 2.7. Data Analysis

The chi-square test was used to compare infection rates in different experimental replicates. The proportional variations of single or mixed infection by FDp strains in the head and body of *E. variegatus* were modelled by logistic GLMs (binomial link) (glm function in package stats). Given the relatively low number of samples, infections by single FDp strains were collapsed into a single category (i.e., “single infection”) when appropriate. Hence, comparisons were performed between single infection and mixed infection in body and head samples separately. Post hoc comparisons were carried out with Tukey’s multiple comparisons correction (*emmeans* functions in the emmeans package and *pairs* function in the graphics package). In some cases, the number of samples in some categories was 0, leading to complete or quasicomplete separation issues in the GLM models. To avoid similar issues, mixed bias-reducing score adjustments were included in the GLM models (*brglmFit* function in the brglm2 package) [47]. The comparisons with expected percentages for single or mixed infection (50% and 100%) were analyzed with an exact binomial test (*binom.test* function in the stats package). All analyses were performed in the statistical software R 4.2.0 [48].

## 3. Results

### 3.1. Mixed Acquisition by Feeding on FD-C- and FD-D-Infected Plants

#### 3.1.1. Phytoplasma Loads in Infected Source Plants

Broad bean plants routinely inoculated to maintain FDp strains were used as source plants for mixed acquisition by feeding. The selected plants were exposed to phytoplasmas during the same inoculation access period and showed similar phytoplasma amounts. Overall, the mean FDp loads measured in each single plant ranged from 4.70 × 10^4^ to 5.10 × 10^5^ (FD-C) and from 4.97 × 10^4^ to 8.10 × 10^5^ (FD-D) phytoplasma GU/ng of plant DNA in the two experimental replicates (Table 1).

#### 3.1.2. Presence and Amount of Phytoplasma Strains in Insect Heads and Bodies

The detection results of phytoplasma strains were similar in the two experimental replicates (chi-square *p* = 0.283) and they are, therefore, pooled together in Table 2.

**Figure 1:**
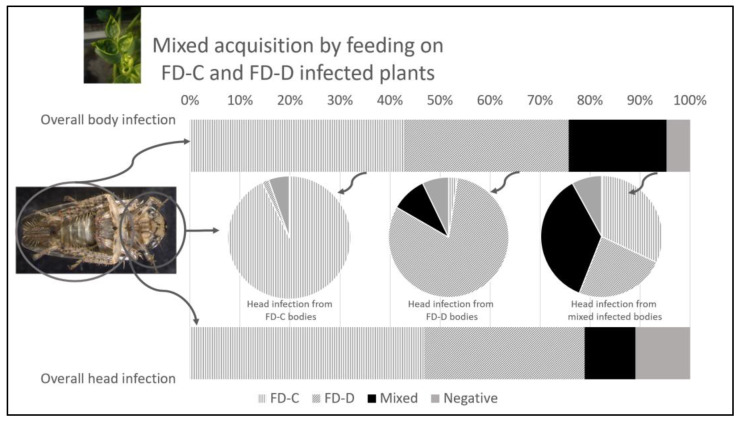
Percentage presence of Flavescence dorée phytoplasmas (FD-C or FD-D strains) detected in insects analyzed following mixed acquisition by feeding on FD-C- and FD-D-infected plants. Upper and lower bars show overall detection in body and head samples, respectively. Pie charts in the middle show phytoplasma detection in head samples obtained from insects with corresponding bodies only infected with FD-C (left chart), with FD-D (central chart), or mixed infected by both strains (right chart). Single-infection condition, irrespective of the strains, is indicated with striped patterns (vertical, FD-C; diagonal, FD-D), whereas full black and grey indicate mixed-infected and negative samples, respectively.

The detection of phytoplasma strains in insects concurrently fed on FD-C and FD-D plants revealed that the single-infection condition, irrespective of the strain, was significantly more frequent than the mixed one, both in the body (75.8% vs. 19.5%, OR = 12.89, z = 8.42, *p*-value < 0.001) and in the head (78.9% vs. 10.2%, OR = 33.09, z = 9.61, *p*-value < 0.001) samples (Table 2; Figure 1 bar charts). Moreover, the observed probability of mixed infection in the body samples (19.5%) was significantly lower than the hypothetical expected probabilities of 100% (z = −16.79, *p*-value < 0.001) and 50% (z = −6.35, *p*-value < 0.001) in the exact binomial tests, supporting the evidence of competition among the strains, which impairs the mixed FD-C/-D-infected condition.

In particular, FD-C was detected in 43.0% and 46.9% of the body and head samples, respectively, whereas FD-D was detected in 32.8% and 32.0% of the cases. Furthermore, 4.7% of the body samples and 10.9% of the heads were negative for the presence of FDp (Table 2; Figure 1, bar charts).

Overall, the mean load of the FD-C strain in the singly infected insects ranged from 2.30 × 10^1^ to 6.43 × 10^4^ (body samples) and from 1.70 × 10^1^ to 2.08 × 10^5^ (head samples) phytoplasma GU/ng of insect DNA in the two experimental replicates (Table 3, Supplementary Table S1). Analogously, the mean load of the FD-D strain in the singly infected insects ranged from 4.60 × 10^1^ to 5.86 × 10^4^ (body samples) and from 2.90 × 10^2^ to 6.00 × 10^5^ (head samples) phytoplasma GU/ng of insect DNA. In the mixed FD-C/-D-infected insects, the mean FD-C load ranged from 6.20 × 10^1^ to 1.47 × 10^4^ (body samples) and from 1.94 × 10^2^ to 2.65 × 10^4^ (head samples) phytoplasma GU/ng of insect DNA, whereas the mean FD-D load ranged from 1.06 × 10^2^ to 1.35 × 10^4^ (body samples) and from 7.80 × 10^1^ to 4.32 × 10^4^ (head samples) phytoplasma GU/ng of insect DNA (Table 3). No significant differences were found in the pathogen load measured in the insect samples.

#### 3.1.3. Insect Infection and Competition between Phytoplasma Strains

To determine the ability of FDp strains to colonize insect heads and eventually to overcome the other phytoplasma isolate, the phytoplasma detection results in head samples were graphed in different pie charts according to the diagnosis results on the corresponding body samples: the bodies singly infected with FD-C or with FD-D, or infected with both strains (Table 2; Figure 1, middle pie charts). When the bodies were infected by a single FDp strain, the corresponding head samples were significantly more frequently colonized by the same isolate, as expected (FD-C 92.7%, OR = 689, z = 5.76, *p*-value < 0.001; FD-D 81%, OR = 174, z = 4.75, *p*-value < 0.001). 

In the cases of the mixed FD-C/-D-infected bodies, 56% of the corresponding head samples were singly infected (32% with FD-C and 24% with FD-D), whereas 36% of the samples still harbored a mixed-strain infection and 8% were negative. The single-strain infection was more frequent than the mixed FD-C/-D one, although not significantly (OR = 2.26, z = 1.41, *p*-value = 0.336).

### 3.2. Mixed Acquisition by Abdominal Microinjection of FD-C and FD-D Suspension

#### 3.2.1. Pathogen Load in Phytoplasma Suspension

The mean FDp load ranged from 7.41 × 10^5^ to 2.18 × 10^6^ (FD-C) and from 4.51 × 10^4^ to 2.08 × 10^6^ (FD-D) in the phytoplasma suspension prepared for injection. Crude extracts were diluted in order to obtain a final amount of 2.8 × 10^5^ in both experimental replicates.

#### 3.2.2. Presence of Phytoplasma Strains in Insect Heads and Bodies

The detection results of phytoplasma strains were similar in the two experimental replicates (chi-square *p* = 0.334) and they were, therefore, pooled together (Table 4).

**Figure 2:**
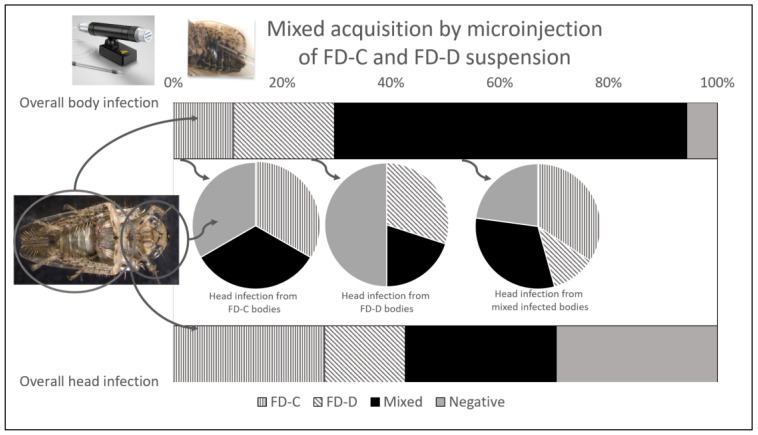
Percentage presence of Flavescence dorée phytoplasmas (FD-C or FD-D strains) detected in insects analyzed following mixed injection of phytoplasma suspension containing equal amount of FD-C and FD-D cells. Upper and lower bars show overall detection in body and head samples, respectively. Pie charts in the middle show phytoplasma detection in head samples obtained from insects with corresponding bodies only infected with FD-C (left chart), with FD-D (central chart), or mixed infected by both strains (right chart). Single-infection condition, irrespective of the strains, is indicated with striped patterns (vertical, FD-C; diagonal, FD-D), whereas full black and grey indicate mixed-infected and negative samples, respectively.

The detection of pathogen strains in insects injected with the FD-C/-D suspension revealed that the mixed-strain infection was significantly more frequent than the single one in the body samples (64.8% vs. 29.6%, OR = 4.38, z = 3.58, *p*-value < 0.001), whereas in the head samples, single infection was the prevalent condition, although not significantly higher than the mixed-strain one (42.6% vs. 27.8%, OR = 1.93, z = 1.60, *p*-value = 0.244) (Table 4; Figure 2 bar charts). Nevertheless, the observed probability of mixed FD-C/-D infection in the body samples (64.8%) was significantly lower than the hypothetical expected probabilities of 100% (z = −14.02, *p*-value < 0.001) in an exact binomial test.

In particular, FD-C was detected in 11.1% and 27.8% of the body and head samples, respectively, whereas FD-D was detected in 18.5% and 14.8% of the cases. Furthermore, 5.6% of the body samples and 29.6% of the heads were negative for the presence of FDp (Table 4; Figure 2, bar charts).

#### 3.2.3. Insect Infection and Competition between Phytoplasma Strains

To determine the ability of FDp strains to colonize insect heads and eventually to overcome the other phytoplasma isolate, the phytoplasma detection results in the head samples were graphed in different pie charts according to the diagnosis results on the corresponding body samples: the bodies only infected with FD-C or with FD-D, or mixed infected by both strains (Table 4; Figure 2, middle pie charts). When the bodies were infected with a single FDp strain, the corresponding head samples were colonized by the same isolate (FD-C 33.3%, FD-D 30%) or by both together (33.3% of the mixed-strain-infected heads with FD-C-infected bodies, and 20% of the mixed-infected heads with FD-D-infected bodies).

In the case of the mixed-strain-infected bodies, 45.7% of the corresponding head samples were singly infected (34.3% with FD-C and 11.4% with FD-D), whereas 31.4% of the samples still harbored mixed-strain infection and 22.9% were negative. The single-strain infection was more frequent than the mixed FD-C/-D one, although not significantly (χ2 = 4.2, df = 2, *p*-value = 0.122).

### 3.3. Phytoplasma Transmission following Mixed Acquisition by Feeding

#### 3.3.1. Presence and Amount of Phytoplasma Strains in Inoculated Plants and in Feeding Media

The detection results of phytoplasma strains were similar in the two experimental replicates and they were, therefore, pooled together and listed in Table 5 and Table 6.

Phytoplasma infection was detected in 58.8% of the inoculated plants and in 36.6% of the analyzed feeding media, irrespective of the strains and of the single-/mixed-strain-infection condition. Five weeks after inoculation, about 50% of the inoculated plants already showed symptoms and were FDp-positive. Despite its lower efficiency in phytoplasma detection, diagnosis from artificial media produced results totally coherent with those from plants inoculated by the corresponding insects. In other words, plants and artificial media inoculated by the same insects either shared the same diagnosis result or displayed a positive plant sample together with a negative feeding medium.

**Figure 3:**
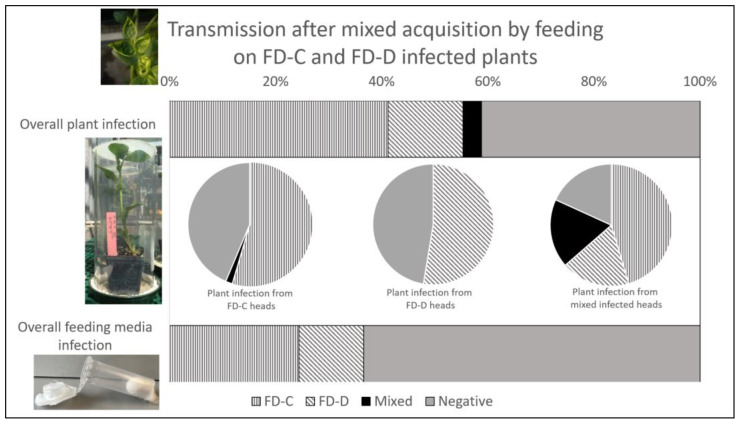
Percentage presence of Flavescence dorée phytoplasmas (FD-C or FD-D strains) detected in plants (upper bar) and feeding media (lower bar) analyzed following inoculation by single insects after mixed acquisition by feeding on FD-C- and FD-D-infected plants. Pie charts in the middle show phytoplasma detection in plants inoculated by insects with corresponding heads only infected with FD-C (left chart), with FD-D (central chart), or mixed-infected by both strains (right chart). Single-strain-infection condition, irrespective of the strain, is indicated with striped patterns (vertical, FD-C; diagonal, FD-D), whereas full black and grey indicate mixed FD-C/-D-infected and negative samples, respectively.

The detection of pathogen strains in plants and feeding media inoculated by insects allowed to feed on FD-C and FD-D plants revealed that the single-infection condition, irrespective of the strain, was more frequent than the mixed one, both in plants (55.3% vs. 3.5%) and in artificial feeding media (36.6% vs. 0%) samples (Table 5 and Table 6; Figure 3 bar charts). In particular, FD-C was detected in 41.2% and 24.4% of the plant and media samples, respectively, whereas FD-D was detected in 14.1% and 12.2% of the cases. Furthermore, 41.2% of the plants and 63.4% of the feeding media samples were negative for the presence of FDp (Table 5 and Table 6; Figure 3, bar charts). Nevertheless, the frequencies of transmission to plants depended on the infection status of the inoculating insects and, therefore, no statistical analysis was run on their overall distributions.

Overall, the mean load of the FD-C strain in the infected broad beans, inoculated by single insects, ranged from 1.10 × 10^2^ to 5.81 × 10^5^ (at 5 wpi) and from 2.80 × 10^2^ to 1.35 × 10^6^ (at 7 wpi) phytoplasma GU/ng of plant DNA in the two experimental replicates (Table 7, Supplementary Table S1). Analogously, the mean loads of the FD-D strain in the infected plants ranged from 4.26 × 10^4^ to 7.03 × 10^5^ (at 5 wpi) and from 1.14 × 10^4^ to 1.08 × 10^6^ (at 7 wpi) phytoplasma GU/ng of plant DNA. No significant differences were found in the pathogen load measured in the plant samples either between strains or among different sampling dates.

#### 3.3.2. Insect Infection and Competition between Phytoplasma Strains

To determine the ability of *E. variegatus* to transmit both FDp strains to plants, the phytoplasma detection results in the plants were graphed in different pie charts according to the diagnosis results on the head samples of the corresponding inoculating insects: the heads only infected with FD-C or with FD-D, or infected by both strains (Table 5; Figure 3, middle pie charts). When heads were infected by a single FDp strain, the inoculated plants were prevalently colonized by the same isolate with similar transmission efficiency (FD-C 54.5%, FD-D 52.6%). Almost all the remaining plants were negative: 43.6% and 47.4% of plants inoculated by insects with FD-C- and FD-D-infected heads, respectively.

In the cases of inoculating insects with mixed-strain-infected heads, 63.6% of the corresponding plants were singly infected (45.4% with FD-C and 18.2% with FD-D), whereas 18.2% of the broad beans harbored mixed-FD-C/-D-strain infections and 18.2% were negative. Again, the single-infection condition was more frequent than the mixed one, although not significantly (OR = 6.33 ± 6.14, z = 1.9, *p*-value = 0.137).

## 4. Discussion

The interaction of two phytoplasma strains of the as-yet-undescribed “*Candidatus* Phytoplasma vitis” species was studied upon the infection of their leafhopper vector. The two strains show differences in genes other than the taxonomically relevant 16S rRNA, but are both transmitted in nature by *Scaphoideus titanus* [34] and are both detected in symptomatic grapevines, although with different prevalence [18,19,49,50]. The interactions of two FDp strains belonging to different 16S rRNA subgroups during the infection of their leafhopper experimental vector *E. variegatus* were analyzed to explore the possibility of interactive or independent transmission.

Cotransmission is common in nature and, due to the complex interaction between plants and pathogens, a synergistic or antagonist interaction may occur as a result of the spatiotemporal order of infection or different multiplication rates [51]. Interestingly, interactions between different microorganisms may alter the outcome of the infection. For example, the establishment of “*Candidatus* Liberibacter asiaticus” is reduced in orange trees previously infected with citrus tristeza virus (CTV) [52]. Competition among isolates of vector-borne plant pathogens may occur for vector proteins involved in transmission, such as stylin or cyclophilin, or through the induction or repression of the vector immune defenses [51]. In particular, the mixed infection of plant pathogens in hemipteran vectors generally impacts epidemiology, as some strains prevail over others [53,54], even if not always [55]. Different CTV isolates can be separated by aphid transmission because individual aphids only transmit a few isolates at once [56,57,58].

Here, strains belonging to two ribosomal subgroups (16SrV-C and -D) of the phytoplasma associated with the Flavescence dorée of grapevine were used to decipher their ability to simultaneously coinfect the insect vector and to be cotransmitted to the host plant. For this purpose, the laboratory hosts *V. faba* and *E. variegatus* were used, as the natural *V. vinifera*/*S. titanus* pathosystem is difficult to handle and requires very long experimental times.

Although *E. variegatus* leafhoppers were forced to feed on both FD-C- and FD-D-infected plants, mixed-strain infection was a rare condition, indicating that competition occurs between isolates during insect colonization. In particular, FD-C colonized salivary glands more efficiently than FD-D in insects with mixed-strain infection. Moreover, the FD-C isolate was also more frequently transmitted to plants than the FD-D one by insects with mixed-strain-infected heads (salivary glands), whereas the single-infected insects transmitted the two strains with similar efficiencies. Transmissions to feeding media, aimed at excluding competition between phytoplasma isolates in the plant, were consistent with transmissions to plants, confirming that FD-C outcompeted FD-D in the inoculation phase. Nevertheless, the quite low number of mixed-strain-infected insects implies that conclusions based on these comparisons must be taken cautiously. Consistently, the FD-C strain clearly prevailed over FD-D during the infection of the experimental plant host, periwinkle [39]. Under field conditions, FD-D is the most prevalent strain [15,17,21,23]. We may hypothesize that the aggressiveness of the FD-C strain in plants [39] often leads to plant death, whereas the more variable and adaptable FD-D isolates [8,15,17,18,19] sustain nonlethal infections. Plants infected by FD-D strains may survive for years and act as sources for vectors and future infections. The FD-D strain would be the most likely to be acquired by insects, on a “first come first served” basis, thus ensuring FD-D prevalence. The general conclusions drawn here for *E. variegatus* can be reasonably extended to the closely related *S. titanus*, the main field vector of the disease. Consistently, among *S. titanus* specimens collected inside and outside of several productive vineyards in Piedmont, reports of mixed infection with FD-C and FD-D isolates have been, overall, very rare [18,21].

*Euscelidius variegatus* can acquire two different phytoplasmas (“*Ca*. P. asteris” chrysanthemum yellows isolate and “*Ca*. P. vitis” FD-C strain) simultaneously, but the two bacteria compete for the colonization of the salivary glands [59]. Conversely, here, competition between FDp strains also occurred during the acquisition phase, probably because the strains belong to the same species and share the same host adhesion and colonization mechanisms. Indeed, to enter insect midgut cells, FDp exploits clathrin-mediated endocytosis, elicited by the phytoplasma variable membrane protein VmpA, an adhesin-like protein abundant on the bacterial cell surface [31]. This internalization mechanism is likely to occur in both FD-C and FD-D strains, as they share the same vector species [19].

The secretion of antimicrobial molecules for direct competition between isolates cannot be excluded, but this aspect is hard to decipher for unculturable pathogens, which can only be studied in alive hosts. Bacteriocins can target even closely related strains of the same species [60], although genes encoding such molecules have not been described in phytoplasma genomes. On the other hand, the AAA+ ATPase protein AP460, a component of the type VI secretion system involved in the control of coinhabiting or competing microbes [61,62], has been described in suppressive strains of “*Ca*. P. mali” [63]. Interestingly, the FDp genome displays a complete protein secretion system with 10 genes involved in this function (*sec*A, *sec*E, *sec*Y, *yid*C, *ffh*, *fts*Y, *dna*J, *dna*K, *grp*E, and *gro*L) [10], which are expressed during host infection [64,65]. Moreover, among AAA+ proteins, eight *fts*H genes are actively transcribed by FDp with a possible role in host adaptation, as most of them are overexpressed in the insect vectors [66].

Finally, the multiplex detection and quantification qPCR method used here was specific for each of the two FDp strains and faster than the conventional Sanger sequencing or restriction fragment length polymorphism techniques. Nevertheless, the few discrepancies in the multiplex diagnosis results might be due to signals below the threshold detection. The pathogen loads were comparable for the two strains in the single-strain-infected insects or plants, indicating that the multiplication dynamics of both isolates were similar in the two hosts. In the mixed-strain-infected insects or plants, the loads of FD-C and FD-D were lower than those measured in single-strain-infected hosts, possibly due to the carrying capacity of the vector.

## 5. Conclusions

Several lines of evidence indicate that competition between FD-C and FD-D strains occurs during insect colonization. Indeed, single infection is the prevalent outcome when insects feed on mixed-strain-infected plants. This may be reasonably explained by competition for resources and/or host active sites, or eventually by the direct antibiosis of one strain against the other, through the secretion of harmful molecules. Additionally, in the insect, FD-C prevails over FD-D during pathogen acquisition, but this prevalence is stronger in periwinkles exposed to mixed-strain infections and results in FD-D displacement [39].

Unveiling the competition between FDp genotypes may identify mild suppressor strains to outcompete the severe ones. The existence of competition among phytoplasma strains encourages further investigations on the exploitation of potential cross-protection, although this perspective must be carefully evaluated for FDp, a quarantine pathogen. However, this knowledge is important for a more detailed picture of the epidemiology of FD in grapevine and for the fine-tuning of disease strategies aiming at reducing insecticide burden in vineyards.

## Figures and Tables

**Table 1:** Amount of Flavescence dorée phytoplasma (FDp) cells (either FD-C or FD-D strains) in each single sample, expressed as mean FDp genome units (GU)/ng of plant DNA ± standard error of the mean (SEM), measured in broad bean plants selected as source of inoculum for “mixed acquisition by feeding” experiments.

# Experiment	FDp Strain	Mean ± SEM (N)
Replicate 1	FD-C	5.52 × 10^4^ ± 7.78 × 10^3^ (2)
	FD-D	5.04 × 10^4^ ± 9.19 × 10^2^ (2)
Replicate 2	FD-C	4.40 × 10^5^ ± 9.90 × 10^4^ (2)
	FD-D	7.10 × 10^4^ ± 1.41 × 10^4^ (2)

**Table 2:** Contingency table describing the presence of Flavescence dorée phytoplasma strains (FD-C or FD-D) detected in insects analyzed following mixed acquisition by feeding on infected plants.

		Body Infection				
		FD-C	FD-D	Mixed FD-C/-D	Negative	Total
Head infection	FD-C	51	1	8	0	60
	FD-D	1	34	6	0	41
	mixed FD-C/-D	0	4	9	0	13
	negative	3	3	2	6	14
	Total	55	42	25	6	128

**Table 3:** Amount of Flavescence dorée phytoplasma (FDp) cells (either FD-C or FD-D strains) in each single sample, expressed as mean FDp genome units (GU)/ng of insect DNA ± standard error of the mean (SEM), measured in insects collected after “Mixed acquisition by feeding” experiments.

Infection Status	FDp Strain	Sample Type	Mean ± SEM
Single-infected samples	FD-C	Bodies	6.40 × 10^3^ ± 1.61 × 10^3^
		Heads	2.43 × 10^4^ ± 4.85 × 10^3^
	FD-D	Bodies	5.08 × 10^3^ ± 1.59 × 10^3^
		Heads	6.81 × 10^4^ ± 1.64 × 10^4^
Mixed-infected samples	FD-C	Bodies	2.14 × 10^3^ ± 8.57 × 10^2^
		Heads	9.97 × 10^3^ ± 3.43 × 10^3^
	FD-D	Bodies	3.44 × 10^3^ ± 9.73 × 10^2^
		Heads	7.38 × 10^3^ ± 3.84 × 10^3^

**Table 4:** Contingency table describing the presence of Flavescence dorée phytoplasma strains (FD-C or FD-D) detected in insects analyzed following mixed acquisition by abdominal microinjection of phytoplasma suspension.

		Body Infection				
		FD-C	FD-D	Mixed FD-C/-D	Negative	Total
Head infection	FD-C	2	0	12	1	15
	FD-D	0	3	4	1	8
	mixed FD-C/-D	2	2	11	0	15
	Negative	2	5	8	1	16
	Total	6	10	35	3	54

**Table 5:** Contingency table describing the presence of Flavescence dorée phytoplasma strains (FD-C or FD-D) cumulatively detected in broad beans at five and seven weeks postinoculation by single insects following mixed acquisition by feeding on FD-C- and FD-D-infected plants. Data of plant infection are organized according to diagnosis on head samples obtained from insects inoculating corresponding plants.

		Head Infection			
		FD-C	FD-D	Mixed FD-C/-D	Total
Plant infection	FD-C	30	0	5	35
	FD-D	0	10	2	12
	mixed FD-C/-D	1	0	2	3
	Negative	24	9	2	35
	Total	55	19	11	85

**Table 6:** Contingency table describing the presence of Flavescence dorée phytoplasma strains (FD-C or FD-D) detected in feeding media inoculated by single insects following mixed acquisition by feeding on FD-C- and FD-D-infected plants. Data of phytoplasma detection in feeding media are organized according to diagnosis on head samples obtained from insects isolated on corresponding media.

		Head Infection			
		FD-C	FD-D	Mixed FD-C/-D	Total
Feeding media detection	FD-C	9	0	1	10
	FD-D	0	3	2	5
	mixed FD-C/-D	0	0	0	0
	Negative	17	7	2	26
	Total	26	10	5	41

**Table 7:** Amount of Flavescence dorée phytoplasma (FDp) cells (either FD-C or FD-D strains) in each single sample, expressed as mean FDp genome units (GU)/ng of plant DNA ± standard error of the mean (SEM), measured in plants collected after five and seven weeks postinoculation (wpi) by insects after “mixed acquisition by feeding” experiment.

Infection Status	FDp Strain	Collection Date	Mean ± SEM
Singly infected samples	FD-C	5 wpi	1.53 × 10^5^ ± 2.75 × 10^4^
		7 wpi	3.12 × 10^5^ ± 7.00 × 10^4^
	FD-D	5 wpi	2.48 × 10^5^ ± 8.04 × 10^4^
		7 wpi	4.71 × 10^5^ ± 1.34 × 10^5^
Mixed-infected samples	FD-C	5 wpi	/
		7 wpi	1.27 × 10^4^
	FD-D	5 wpi	/
		7 wpi	9.32 × 10^3^

## Data Availability

Data is contained within the article or supplementary material.

## Supplementary Materials

The following supporting information can be downloaded at: https://www.mdpi.com/article/10.3390/insects14070575/s1, Table S1: Amount of phytoplasma cells measured in each single sample.
