# Supplementary material for: Competition among Flavescence Dorée Phytoplasma Strains in the Experimental Insect Vector Euscelidius variegatus
Source: Insects. 2023 Jun 23;14(7):575. doi: 10.3390/insects14070575 (PMC10380400; doi:10.3390/insects14070575)
Supplement: Supplementary file 1 [file insects-14-00575-s001.zip › insects-2449556-supplementary.pdf]

Amount of phytoplasma cells measured in each single sample

Amount of Flavescence dorée phytoplasma (FDp) cells (either FD-C or FD-D strains) in each single sample, expressed as FDp Genome Units (GU)/ng of insect DNA, measured in insects collected after 'Mixed acquisition by feeding' experiments. SEM: standard error of the mean

Single infected insects				Mixed infected insects			
FD-C Bodies	FD-C Heads	FD-D Bodies	FD-D Heads	FD-C Bodies	FD-C Heads	FD-D Bodies	FD-D Heads
2,67E+03	3,09E+04	6,98E+02	1,34E+04	9,76E+02	2,17E+04	1,13E+04	1,37E+04
1,70E+04	3,47E+02	1,76E+04	4,04E+05	1,71E+02	1,87E+03	1,35E+04	4,32E+04
9,63E+02	7,09E+03	5,86E+04	3,74E+04	6,44E+03	2,65E+04	5,71E+03	3,01E+03
6,77E+03	8,41E+04	2,60E+04	6,00E+05	1,08E+03	2,52E+03	5,89E+03	6,71E+03
8,59E+03	1,34E+05	1,38E+04	2,74E+04	1,70E+03	1,37E+04	1,03E+04	3,80E+02
6,43E+04	3,74E+02	4,45E+03	1,87E+05	1,47E+04	2,57E+04	1,13E+04	9,92E+03
2,40E+03	8,61E+03	9,74E+03	1,32E+04	1,41E+02	1,94E+02	7,13E+02	5,05E+02
3,99E+02	6,20E+03	2,67E+03	3,28E+04	2,38E+02	2,16E+03	2,70E+02	7,80E+01
4,15E+03	2,59E+03	3,71E+03	1,06E+05	5,87E+02	2,67E+03	7,66E+02	3,22E+03
1,68E+04	6,53E+04	9,30E+02	5,26E+04	6,20E+01	2,67E+03	2,39E+02	2,28E+02
3,26E+03	9,10E+03	4,56E+02	3,69E+03	2,23E+03		3,21E+02	2,28E+02
2,30E+01	5,77E+04	5,84E+03	8,89E+02	8,25E+02		4,22E+02	
1,55E+04	7,46E+04	7,40E+01	1,56E+04	1,29E+03		2,10E+03	
4,11E+03	2,55E+04	3,32E+03	1,24E+04	1,68E+02		1,04E+03	
4,85E+04	5,50E+04	4,60E+01	4,06E+03	9,11E+02		4,15E+02	
1,03E+03	2,91E+04	1,21E+03	1,66E+04	9,91E+03		4,96E+03	
4,04E+03	9,60E+01	1,90E+03	3,55E+03	1,20E+02		2,68E+02	
4,59E+03	4,48E+04	2,94E+03	7,45E+03	1,04E+03		1,73E+03	
5,14E+03	3,29E+04	9,14E+03	2,90E+02	1,46E+02		1,06E+02	
6,02E+03	8,70E+01	1,30E+02	3,87E+04	7,30E+01		4,27E+02	
2,89E+03	1,04E+02	3,29E+02	5,35E+03			4,84E+02	
6,61E+02	7,39E+04	3,80E+03	3,69E+03				
4,76E+02	5,53E+03	6,10E+02	1,08E+03				
1,93E+03	1,60E+04	2,02E+03	5,95E+03				
5,77E+02	9,83E+02	2,14E+03	4,05E+04				
1,50E+03	2,16E+03	6,90E+03	1,50E+04				
4,44E+03	1,87E+04	7,10E+03	1,19E+05				
2,42E+04	1,75E+04	5,70E+02	5,55E+03				
4,71E+03	7,34E+04	1,22E+04	1,68E+05				
1,80E+03	1,58E+03	2,44E+02	1,39E+05				
2,22E+03	1,79E+04	2,72E+03	2,94E+03				
1,84E+02	6,10E+01	1,08E+02	8,75E+04				
1,78E+03	1,12E+04	3,08E+02	1,49E+05				
9,54E+03	2,38E+04	9,45E+01	1,18E+05				
3,11E+03	1,76E+04	1,18E+02	8,75E+04				
3,63E+02	5,26E+02	1,74E+03	7,95E+04				
1,96E+03	1,95E+03	3,54E+03	4,00E+04				
2,09E+03	1,53E+04	2,62E+02	1,44E+04				
1,35E+03	7,80E+02	5,35E+01	1,12E+03				
7,67E+03	2,22E+03	8,60E+01	4,92E+03				
1,30E+04	6,41E+03	1,44E+02	4,92E+03				
4,36E+03	3,20E+04						
1,81E+03	1,45E+02						
2,22E+03	9,80E+03						
4,08E+03	1,36E+04						
1,20E+03	9,45E+03						
7,60E+02	4,43E+03						
9,65E+02	5,30E+01						
8,00E+03	9,33E+03						
4,04E+02	8,80E+01						
4,41E+01	4,28E+03						
	2,41E+04						
	2,78E+02						
	1,70E+01						
	2,08E+05						
	3,55E+04						
	6,75E+04						
	1,24E+04						

	Single infected insects				Mixed infected insects			
	FD-C Bodies	FD-C Heads	FD-D Bodies	FD-D Heads	FD-C Bodies	FD-C Heads	FD-D Bodies	FD-D Heads
MEAN	6,40E+03	2,43E+04	5,08E+03	6,51E+04	2,14E+03	9,97E+03	3,44E+03	7,38E+03
DEV STANDARD	1,15E+04	3,69E+04	1,02E+04	1,15E+05	3,83E+03	1,08E+04	4,46E+03	1,27E+04
SEM	1,61E+03	4,85E+03	1,59E+03	1,79E+04	8,57E+02	3,43E+03	9,73E+02	3,84E+03

Amount of Flavescence dorée phytoplasma (FDp) cells (either FD-C or FD-D strains) in each single sample, expressed as FDp Genome Units (GU)/ng of plant DNA, measured in plants collected after five and seven weeks post inoculation (wpi) by insects after 'mixed acquisi-tion by feeding' experiment. SEM: standard error of the mean

Single infected plant_5wpi		Single infected plant_7wpi		Mixed infected plant_7wpi	
FD-C	FD-D	FD-C	FD-D	FD-C	FD-D
1,77E+05	1,36E+05	1,50E+05	2,54E+05	1,27E+04	9,32E+03
3,90E+03	1,12E+05	3,90E+01	8,21E+05		
5,81E+05	4,26E+04	6,90E+01	1,04E+05		
7,07E+04	3,54E+05	2,80E+01	2,21E+05		
2,39E+05	7,03E+05	2,75E+05	1,08E+06		
1,86E+05	4,19E+05	3,43E+05	6,70E+05		
1,45E+05	7,97E+04	3,20E+01	1,14E+04		
4,79E+03	1,34E+05	2,23E+05	6,05E+05		
1,18E+05		8,67E+05			
1,07E+05		3,50E+01			
2,59E+05		1,35E+06			
1,03E+05		5,27E+05			
1,44E+05		4,94E+05			
3,27E+05		6,30E+01			
1,60E+02		2,01E+02			
2,65E+05		1,27E+02			
1,10E+02		3,52E+05			
6,21E+04		8,60E+04			
4,35E+04		5,62E+05			
2,92E+02		5,57E+05			
1,36E+05		2,76E+02			
1,91E+05		7,72E+02			
2,98E+05		3,72E+02			
1,99E+05		3,59E+05			
		7,88E+02			
		1,88E+05			
		8,30E+01			
		9,07E+05			
		1,40E+06			
		7,70E+05			
		1,61E+03			
		5,70E+05			

	Single infected plant_5wpi		Single infected plant_7wpi		Mixed infected plant_7wpi	
	FD-C	FD-D	FD-C	FD-D	FD-C	FD-D
MEAN	1,53E+05	2,48E+05	3,12E+05	4,71E+05	1,27E+04	9,32E+03
DEV STANDARD	1,35E+05	2,27E+05	3,96E+05	3,79E+05		
SEM	2,75E+04	8,04E+04	7,01E+04	1,34E+05		
